# Association between atherogenic index of plasma and cognitive impairment in middle-aged and older adults: results from CHARLS

**DOI:** 10.3389/fnagi.2025.1506973

**Published:** 2025-02-26

**Authors:** Juan Zhou, Han Han, Weimin Bai

**Affiliations:** ^1^Department of Endocrinology, The People’s Hospital of Danyang, Affiliated Danyang Hospital of Nantong University, Danyang, Jiangsu, China; ^2^Department of Emergency, Henan Provincial People’s Hospital, People’s Hospital of Zhengzhou University, People’s Hospital of Henan University, Zhengzhou, China

**Keywords:** atherogenic index of plasma, cognitive impairment, CHARLS, triglycerides, high-density lipoprotein cholesterol

## Abstract

**Background:**

The atherogenic index of plasma (AIP) has been proposed as a novel biomarker predictor for dyslipidemia and has been linked to various diseases. In this study, we explored the relationship between AIP levels and cognitive impairment in a middle-aged and older population.

**Methods:**

This study utilized data from the China Health and Retirement Longitudinal Study (CHARLS) for 7,918 individuals aged 45 and older. The AIP was calculated as the logarithmic ratio of triglycerides to high-density lipoprotein cholesterol. To assess the relationship between the AIP and cognitive impairment, logistic regression models were employed, while restricted cubic spline analysis was conducted to explore potential non-linear associations between AIP levels and cognitive impairment.

**Results:**

The study participants had a mean age of 58.4 ± 8.8 years, and 49.1% were female. From 2011 to 2018, 2,911 participants (36.8%) developed cognitive impairment. After adjusting for potential confounders, the AIP was found to be significantly associated with cognitive impairment. In particular, participants in the higher AIP quartiles (Q2: odds ratio [OR]: 1.45, 95% confidence interval [CI]: 1.24–1.69, *P* < 0.001, Q3: OR: 1.63, 95% CI: 1.40–1.91, *P* < 0.001, and Q4: OR: 1.68, 95% CI: 1.43–1.98, *P* < 0.001) showed an increased risk of cognitive impairment compared to those in the lowest quartile (Q1). Additionally, a non-linear relationship was observed between AIP levels and cognitive impairment risk (P for nonlinear < 0.001).

**Conclusion:**

The study finds that elevated AIP levels are linked to an increased risk of cognitive impairment in middle-aged and older adults, suggesting that managing dyslipidemia could help reduce this risk.

## 1 Introduction

Cognitive impairment is a major contributor to disability and has become a critical global public health concern (Alzheimer’s Association, 2015; Llibre Rodriguez et al., 2008; Roberts and Knopman, 2013). According to the World Health Organization, cognitive impairment represents a substantial threat to healthy aging among middle-aged and older populations (Ping et al., 2020). As of 2010, it was estimated that approximately 38.77 million individuals in China were living with mild cognitive impairment, while 15.07 million were affected by dementia (Jia et al., 2020). Despite its significance, there remains a lack of effective treatments for cognitive impairment. Therefore, early identification and timely intervention are essential for mitigating the risk and progression of cognitive impairment.

Numerous studies have demonstrated that metabolic disorders, such as dyslipidemia, are significant risk factors for cognitive impairment (Bai et al., 2024; Farr et al., 2008; Feinkohl et al., 2019; Kivipelto and Solomon, 2006; Ma et al., 2017). Several lipid markers, including total cholesterol, triglycerides (TG), and high-density lipoprotein cholesterol (HDL-C), have been associated with the risk of cognitive impairment (Bai et al., 2024; Feinkohl et al., 2019; Reynolds et al., 2010; Shao et al., 2017; Whitmer et al., 2005); however, their predictive accuracy remains limited. The atherogenic index of plasma (AIP), defined as the logarithmic ratio of TG to HDL-C, is a sensitive marker reflecting the lipoprotein profile of plasma lipids and is considered a more robust indicator of dyslipidemia than either TG or HDL-C alone (Dobiásová and Frohlich, 2001; McLaughlin et al., 2003; Onat et al., 2010; Zhu et al., 2015). The AIP has been found to be significantly associated with various diseases, including cardiovascular disease, diabetes, and other metabolic disorders (Jiang et al., 2024; Qu et al., 2024). However, the relationship between AIP and cognitive impairment remains uncertain. Given its cost-effectiveness and ease of accessibility as a routine clinical measure, the AIP stands out as a simple, reliable, and affordable tool. Its straightforward implementation offers a clear advantage over more complex and costly methods, making it an efficient option for routine clinical practice.

Thus, based on data from the China Health and Retirement Longitudinal Study (CHARLS), this study investigates the relationship between the AIP and cognitive impairment in middle-aged and older individuals to provide population-based evidence for the association between the AIP and cognitive impairment. We hypothesize that elevated AIP, indicative of dyslipidemia marked by elevated TG and reduced HDL-C levels, may contribute to cognitive impairment by promoting inflammation, oxidative stress, and vascular damage, all of which are increasingly recognized as central mechanisms in the pathogenesis of cognitive impairment.

## 2 Materials and methods

### 2.1 Study population

**FIGURE 1:**
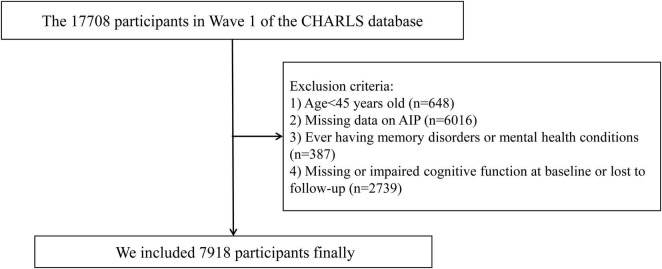
Study flowchart. CHARLS, China Health and Retirement Longitudinal Study; AIP, atherogenic index of plasma.

All participants in this study were drawn from the CHARLS, a nationally representative cohort study that began in 2011. The cohort focuses on individuals aged 45 and older in China.^1^ Using a multi-stage probability sampling technique, participants were selected from 150 counties (districts) and 450 villages (urban) communities across China. Follow-up assessments have since been conducted every 2–3 years to track the health status of participants. Details of the study methodology have been described previously (Zhao et al., 2014). In the present study, we used data from the 2011 and 2018 waves of CHARLS, with the 2011 wave serving as the baseline. In the first wave of CHARLS, 17,708 participants were initially recruited. Participants were excluded for the following reasons: (1) age < 45 years (*n* = 648); (2) missing data on AIP (*n* = 6,016); (3) diagnosed memory disorders or mental health conditions (*n* = 387); and (4) missing or impaired cognitive function at baseline or lost to follow-up (*n* = 2,739). The missing data on AIP were assumed to be Missing at Random (MAR). As a result, 7,918 participants were included in the final analysis. Details of the inclusion/exclusion process are presented in Figure 1.

The collection of CHARLS data received ethical approval from the Biomedical Ethics Review Board of Peking University (IRB00001052-11015), and all participants provided written informed consent.

### 2.2 Measurement of cognitive function and AIP

In the 2011 and 2018 follow-up surveys, the participants’ cognitive function was assessed using tests of episodic memory and mental intactness using methods similar to those used in the American Health and Retirement Study (Zhao et al., 2014). For episodic memory, participants were first asked to immediately recall ten words read aloud by the interviewer. Approximately 4 min later, they were asked to recall these words again to assess delayed recall. Episodic memory was evaluated based on the average score of the immediate and delayed recall tasks, with possible scores ranging from 0 to 10. To assess mental intactness, participants completed a series of tasks, including accurately drawing a specific figure, answering questions about the current date, season, and day of the week, and performing a series of subtraction tasks (subtracting 7 from 100 five times consecutively). Each correct answer was awarded one point, with a maximum possible score of 11 (Ding and He, 2021; Lu et al., 2021). The sum of these scores represented the participants’ overall cognitive function, with total scores ranging from 0 to 21, where higher scores indicated better cognitive performance. Cognitive impairment was defined as a score that was 1.0 standard deviation or more below the mean cognitive function score based on previous studies (Bai et al., 2021; Chai et al., 2024; Jak et al., 2009).

Blood lipid levels were measured using enzymatic colorimetric assays (Chen et al., 2022). The AIP was calculated using the formula AIP = log10 (TG/HDL-C), where TG and HDL-C are measured in mg/dL. Participants were then categorized into four quartiles based on their AIP values: Q1 (<0.126), Q2 (≥0.126–0.337), Q3 (≥0.337–0.564), and Q4 (≥0.564).

### 2.3 Data collection and definitions

The following categories of variables were investigated in this study: (1) sociodemographic factors (age, gender, marital status, residence, and education level; (2) anthropometric measurements (body mass index [BMI]); (3) health behaviors (smoking [never or former vs. current] and drinking status [never or former vs. current]); (4) medical history (hypertension, diabetes, dyslipidemia, stroke, coronary heart disease [CHD], and chronic diseases); and (5) laboratory measurements (total cholesterol [TC], TG, HDL-C, low-density lipoprotein cholesterol [LDL-C], C-reactive protein [CRP], fasting blood glucose [FBG], and glycated hemoglobin [HbA1c]). Diabetes was defined as FBG ≥ 126 mg/dL, HbA1c ≥ 6.5%, and/or self-reported physician-diagnosed diabetes, and/or the use of hypoglycemic agents (ElSayed et al., 2023). Hypertension was defined as self-reported hypertension, treatment for hypertension, and/or a systolic blood pressure (SBP) ≥ 140 mmHg or diastolic blood pressure (DBP) ≥90 mmHg. Dyslipidemia was identified through self-reported physician diagnosis and/or current use of lipid-lowering medications, and/or meeting the following criteria: TC ≥ 240 mg/dL, TG ≥ 150 mg/dL, HDL-C < 40 mg/dL, or LDL-C ≥ 160 mg/dL (Zhou et al., 2022). Missing data for key variables (e.g., lipid profiles and cognitive scores) were excluded during the participant selection process.

### 2.4 Statistical analysis

Descriptive statistics were used to present the data, with results expressed as the mean ± standard deviation, median (interquartile range), or frequencies and percentages, depending on the variable type. Categorical variables were analyzed using the chi-square test, whereas continuous variables were assessed using one-way analysis of variance (ANOVA) or Kruskal-Wallis tests for non-normally distributed data.

The relationship between the AIP and cognitive impairment was analyzed using logistic regression, with results reported as adjusted odds ratios (OR) and 95% confidence intervals (CI). Covariates were selected based on existing literature and clinical judgment (Bai et al., 2024; Feinkohl et al., 2019; Morrow et al., 1968; Reynolds et al., 2010; Shao et al., 2017). AIP was examined both as a continuous variable and categorized into quartiles to explore the relationship with cognitive impairment. In the analysis, Model 1 involved univariate logistic regression, Model 2 was adjusted for age and gender, and Model 3 included adjustments for a broader set of variables, including age, gender, BMI, residence, education level, marital status, health, smoking and drinking status, hypertension, diabetes, chronic diseases, TC, LDL-C, FBG, HbAlc, and cognitive function score in 2011. Restricted cubic splines were also utilized to explore the shape of the association between AIP levels and cognitive impairment, with the median AIP value serving as the reference for comparison.

To explore the association between the AIP and cognitive impairment risk across different groups, subgroup analyses were performed based on age (45–60 years and ≥60 years), gender (male and female), BMI (<24 kg/m^2^ and ≥24 kg/m^2^), residence (urban and rural), diabetes (yes and no), and dyslipidemia (yes and no). These statistical analyses were carried out using R version 4.2.2 (R Foundation for Statistical Computing, Vienna, Austria). A two-sided *P*-value of less than 0.05 was considered indicative of statistical significance.

## 3 Results

### 3.1 Baseline characteristics

The study included a total of 7,918 participants, with a mean baseline age of 58.4 ± 8.8 years. Of the participants, 49.1% (3,886) were female. Participants in the higher AIP quartiles were generally younger, more likely to be married, lived in urban areas, and had higher levels of education compared to those in the lowest quartile. They also had lower rates of current smoking but higher prevalence of hypertension, diabetes, dyslipidemia, stroke, and CHD. Furthermore, individuals in higher AIP quartiles exhibited elevated levels of TC, TG, FBG, HbA1c, and CRP, while HDL-C levels were lower. The baseline characteristics of the study population are detailed in Table 1.

**TABLE 1:** Baseline characteristics of participants categorized by AIP quartiles.

Characteristic	Overall (*n* = 7918)	AIP				
		Quartile 1 (*n* = 1980)	Quartile 2 (*n* = 1978)	Quartile 3 (*n* = 1980)	Quartile 4 (*n* = 1980)	*P*-value
Age, mean ± SD, years	58.4 ± 8.8	58.9 ± 9.3	58.7 ± 9.0	58.4 ± 8.7	57.7 ± 8.2	<0.001
Female, *n* (%)	3886 (49.1)	876 (44.2)	970 (49.0)	1054 (53.2)	986 (49.8)	<0.001
BMI, kg/m^2^	24.32 (31.27)	22.05 (3.29)	25.39 (61.04)	24.53 (11.52)	25.37 (3.90)	0.004
Marital status, *n* (%)						0.042
Married	7118 (89.9)	1759 (88.8)	1783 (90.1)	1767 (89.2)	1809 (91.4)	
Others	800 (10.1)	221 (11.2)	195 (9.9)	213 (10.8)	171 (8.6)	
Residence, *n* (%)						<0.001
Rural	4753 (60.0)	1352 (68.3)	1223 (61.8)	1134 (57.3)	1044 (52.7)	
Urban	3165 (40.0)	628 (31.7)	755 (38.2)	846 (42.7)	936 (47.3)	
Education level, *n* (%)						<0.001
No formal education	3105 (39.2)	848 (42.9)	775 (39.2)	769 (38.9)	713 (36.0)	
Primary school	1911 (24.2)	475 (24.0)	487 (24.6)	477 (24.1)	472 (23.9)	
Middle or high school	1868 (23.6)	454 (23.0)	463 (23.4)	464 (23.5)	487 (24.6)	
College or above	1027 (13.0)	201 (10.2)	251 (12.7)	268 (13.5)	307 (15.5)	
Health, *n* (%)						0.001
Poor	278 (3.5)	53 (2.7)	69 (3.5)	81 (4.1)	75 (3.8)	
Fair	1653 (20.9)	412 (20.8)	382 (19.3)	447 (22.6)	412 (20.8)	
Good	4115 (52.0)	1069 (54.0)	1066 (53.9)	1001 (50.6)	979 (49.4)	
Very good and above	1872 (23.7)	446 (22.5)	461 (23.3)	451 (22.8)	514 (25.9)	
Smoking status, *n* (%)						0.001
Never or former	5346 (67.5)	1285 (64.9)	1307 (66.1)	1371 (69.2)	1383 (69.8)	
Current	2572 (32.5)	695 (35.1)	671 (33.9)	609 (30.8)	597 (30.2)	
Drinking status, *n* (%)						<0.001
Never or former	5122 (64.7)	1148 (58.0)	1292 (65.3)	1355 (68.4)	1327 (67.0)	
Current	2796 (35.3)	832 (42.0)	686 (34.7)	625 (31.6)	653 (33.0)	
Hypertension, *n* (%)	3636 (46.0)	755 (38.2)	841 (42.6)	954 (48.3)	1086 (54.9)	<0.001
Diabetes, *n* (%)	1183 (14.9)	167 (8.4)	218 (11.0)	296 (14.9)	502 (25.4)	<0.001
Dyslipidemia, *n* (%)	4102 (51.8)	682 (34.4)	837 (42.3)	1039 (52.5)	1544 (78.0)	<0.001
CHD, *n* (%)	996 (12.6)	178 (9.0)	211 (10.7)	276 (14.0)	331 (16.8)	<0.001
Stroke, *n* (%)	179 (2.3)	35 (1.8)	45 (2.3)	39 (2.0)	60 (3.0)	0.041
Chronic diseases, *n* (%)	5448 (68.8)	1269 (64.1)	1335 (67.5)	1417 (71.6)	1427 (72.1)	<0.001
TC, mean ± SD, mg/dl	193.19 ± 38.01	187.29 ± 34.29	189.79 ± 36.97	193.80 ± 37.22	201.85 ± 41.61	<0.001
TG, median (IQR), mg/dl	107.09 (75.23, 156.65)	62.01 (52.23, 71.74)	90.29 (78.77, 104.56)	127.15 (110.51, 146.25)	212.03 (173.21, 282.16)	<0.001
HDL-C, mean ± SD, mg/dl	50.66 ± 15.10	66.37 ± 13.87	53.59 ± 9.97	46.14 ± 8.66	36.54 ± 8.38	<0.001
LDL-C, mean ± SD, mg/dl	116.42 ± 34.70	111.00 ± 30.08	120.05 ± 33.32	123.28 ± 33.90	111.31 ± 39.25	<0.001
FBG, mean ± SD, mg/dl	110.18 ± 7.06	101.60 ± 22.45	105.42 ± 29.42	109.35 ± 36.09	124.36 ± 50.26	<0.001
HbAlc, mean ± SD, %	5.26 ± 0.81	5.12 ± 0.60	5.20 ± 0.68	5.26 ± 0.80	5.46 ± 1.05	<0.001
CRP, median (IQR), mg/l	1.03 (0.55, 2.14)	0.76 (0.44, 1.72)	0.90 (0.52, 1.86)	1.12 (0.60, 2.31)	1.33 (0.72, 2.68)	<0.001
Cognitive function in 2011	10.90 (3.24)	11.84 (3.36)	10.80 (3.17)	10.76 (3.17)	10.18 (3.02)	<0.001
Cognitive function in 2018	7.72 (5.50)	8.93 (5.65)	7.93 (5.55)	7.16 (5.37)	6.85 (5.20)	<0.001

AIP, atherogenic index of plasma; BMI, body mass index; CHD, coronary heart disease; CRP, C-reactive protein; FBG, fasting blood glucose; HbAlc, glycated hemoglobin; HDL-C, high-density lipoprotein cholesterol; IQR, inter quartile range; LDL-C, low-density lipoprotein cholesterol; SD, standard deviation; TC, total cholesterol; TG, triglycerides.

### 3.2 Association between AIP and cognitive impairment

**FIGURE 2:**
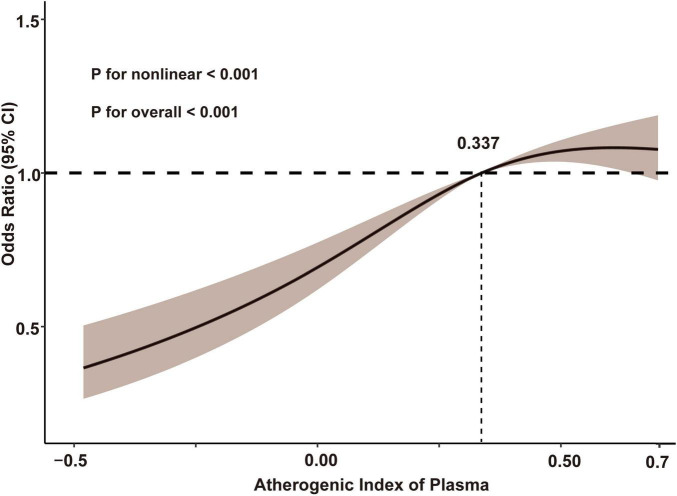
Restricted cubic spline analysis with multivariate-adjusted associations between AIP and cognitive impairment. Age, gender, body mass index, marital status, residence, educational level, health, smoking status, drinking status, chronic diseases, hypertension, diabetes, low-density lipoprotein cholesterol, total cholesterol, fasting blood glucose, glycated hemoglobin, and cognitive function score in 2011 were adjusted. AIP, atherogenic index of plasma.

The cognitive function assessment conducted during the 2018 follow-up produced an average score of 7.72 ± 5.50. The prevalence of cognitive impairment across the AIP quartiles was as follows: Q1: 31.3% (619/1980), Q2: 36.8% (728/1978), Q3: 39.4% (780/1980), and Q4: 39.6% (784/1980). The findings revealed a strong positive association between the AIP and cognitive impairment in both the crude and fully adjusted models. After adjusting for potential confounders, participants in the higher AIP quartiles (Q2, Q3, and Q4) had an increased risk of cognitive impairment compared to those in Q1 (Q2: OR: 1.45, 95% CI: 1.24–1.69, *P* < 0.001; Q3: OR: 1.63, 95% CI: 1.40–1.91, *P* < 0.001; Q4: OR: 1.68, 95% CI: 1.43–1.98, *P* < 0.001, respectively). Additionally, each one-unit rise in the AIP was associated with a 74% greater likelihood of cognitive impairment (OR: 1.74, 95% CI: 1.45–2.09, *P* < 0.001) (Table 2). Multivariable-adjusted restricted cubic spline analysis also confirmed a non-linear relationship between AIP levels and cognitive impairment (overall: *P* < 0.001; non-linear: *P* < 0.001). The risk of cognitive impairment clearly increased with higher AIP levels below the median value of 0.337. However, above this threshold, the rate of increase in the risk of cognitive impairment slightly slowed (Figure 2).

**TABLE 2:** The association between AIP and the risk of cognitive impairment.

Categories	Event, *n* (%)	Model 1^a^		Model 2^b^		Model 3^c^	
		OR (95% CI)	*P*-value	OR (95% CI)	*P*-value	OR (95% CI)	*P*-value
Per 1 unit increase	2911 (36.8%)	1.39 (1.22–1.59)	<0.001	1.58 (1.37–1.82)	<0.001	1.74 (1.45–2.09)	<0.001
Quartile 1	619 (31.3%)	Ref.		Ref.		Ref.	
Quartile 2	728 (36.8%)	1.28 (1.22–1.46)	<0.001	1.31 (1.14–1.51)	0.001	1.45 (1.24–1.69)	<0.001
Quartile 3	780 (39.4%)	1.43 (1.26–1.64)	<0.001	1.50 (1.30–1.72)	<0.001	1.63 (1.40–1.91)	<0.001
Quartile 4	784 (39.6%)	1.44 (1.27–1.65)	<0.001	1.61 (1.40–1.84)	<0.001	1.68 (1.43–1.98)	<0.001
Pfor trend			<0.001		<0.001		<0.001

AIP, atherogenic index of plasma; CI, confidence interval; OR, odds ratio.

*^a^*Unadjusted model.

*^b^*Adjusted for age and gender.

*^c^*Adjusted for age, gender, body mass index, marital status, residence, educational level, health, smoking status, drinking status, chronic diseases, hypertension, diabetes, low-density lipoprotein cholesterol, total cholesterol, fasting blood glucose, glycated hemoglobin, and cognitive function score in 2011.

### 3.3 Subgroup analysis

Stratified subgroup analyses based on factors such as age, gender, BMI, residence, diabetes, and dyslipidemia consistently produced similar results, with no significant interaction effects observed (P values for interaction > 0.05). Across all subgroups, participants with higher AIP levels had a significantly higher risk of cognitive impairment (Table 3).

**TABLE 3:** Subgroup and interaction analyses of the association between AIP and cognitive impairment across various subgroups.

Sub groups	AIP	Event (%)	OR (95% CI)	*P*-value		AIP	Event (%)	OR (95% CI)	*P*-value	P-interaction
**Age**										0.818
<60 years	Q1	279 (24.7)	Ref.		≥60 years	Q1	355 (41.8)	Ref.		
	Q2	329 (29.3)	1.42 (1.15–1.76)	0.001		Q2	412 (48.1)	1.54 (1.22–1.93)	<0.001	
	Q3	358 (31.5)	1.57 (1.26–1.94)	<0.001		Q3	442 (52.3)	1.97 (1.57–2.49)	<0.001	
	Q4	421 (34.4)	1.84 (1.47–2.31)	<0.001		Q4	392 (51.9)	1.93 (1.51–2.47)	<0.001	
**Gender**										0.548
Female	Q1	282 (32.2)	Ref.		Male	Q1	352 (31.9)	Ref.		
	Q2	381 (39.3)	1.44 (1.15–1.81)	0.001		Q2	360 (35.7)	1.41 (1.14–1.74)	0.001	
	Q3	463 (43.9)	1.82 (1.46–2.29)	<0.001		Q3	337 (36.4)	1.49 (1.20–1.85)	<0.001	
	Q4	445 (45.1)	1.87 (1.47–2.39)	<0.001		Q4	368 (37.0)	1.64 (1.31–2.06)	<0.001	
**BMI**										0.058
<24 kg/m^2^	Q1	444 (32.2)	Ref.		≥24 kg/m^2^	Q1	116 (29.0)	Ref.		
	Q2	441 (40.0)	1.52 (1.27–1.83)	<0.001		Q2	219 (33.2)	1.25 (0.93–1.67)	0.142	
	Q3	392 (44.6)	1.90 (1.56–2.30)	<0.001		Q3	309 (35.5)	1.35 (1.02–1.79)	0.037	
	Q4	293 (44.9)	2.09 (1.67–2.62)	<0.001		Q4	408 (38.2)	1.50 (1.13–1.99)	0.005	
**Residence**										0.115
Urban	Q1	194 (30.9)	Ref.		Rural	Q1	440 (32.5)	Ref.		
	Q2	273 (36.2)	1.31 (1.01–1.71)	0.043		Q2	468 (38.3)	1.52 (1.25–1.84)	<0.001	
	Q3	339 (40.1)	1.49 (1.15–1.94)	0.003		Q3	461 (40.6)	1.77 (1.45–2.15)	<0.001	
	Q4	367 (39.2)	1.43 (1.10–1.86)	0.008		Q4	446 (42.7)	2.06 (1.67–2.53)	<0.001	
**Diabetes**										0.310
No	Q1	580 (32.0)	Ref.		Yes	Q1	54 (32.3)	Ref.		
	Q2	635 (34.1)	1.39 (1.18–1.64)	<0.001		Q2	106 (48.6)	2.10 (1.30–3.43)	0.003	
	Q3	668 (39.7)	1.66 (1.40–1.95)	<0.001		Q3	132 (44.6)	1.86 (1.17–2.99)	0.009	
	Q4	587 (39.7)	1.72 (1.44–2.05)	<0.001		Q4	226 (45.0)	2.22 (1.40–3.53)	<0.001	
**Dyslipidemia**										0.300
No	Q1	411 (31.7)	Ref.		Yes	Q1	223 (32.7)	Ref.		
	Q2	422 (37.0)	1.46 (1.20–1.78)	<0.001		Q2	319 (38.1)	1.41 (1.10–1.81)	0.006	
	Q3	370 (39.3)	1.51 (1.23–1.86)	<0.001		Q3	430 (41.4)	1.81 (1.42–2.30)	<0.001	
	Q4	187 (42.9)	1.75 (1.32–2.30)	<0.001		Q4	626 (40.5)	1.80 (1.42–2.27)	<0.001	

Model adjusted for age, gender, body mass index, marital status, residence, educational level, health, smoking status, drinking status, chronic diseases, hypertension, diabetes, low-density lipoprotein cholesterol, total cholesterol, fasting blood glucose, glycated hemoglobin, and cognitive function score in 2011. AIP, atherogenic index of plasma; BMI, body mass index; OR, odds ratios; CI, confidence intervals.

## 4 Discussion

In this nationwide longitudinal cohort study of middle-aged and older individuals undergoing health examinations, we investigated the association between the AIP and cognitive impairment. Our findings revealed a significant positive correlation between higher baseline AIP levels and an increased risk of developing cognitive impairment, as well as a non-linear relationship between the AIP and cognitive impairment. To the best of our knowledge, this is one of the first studies to explore this association, suggesting that the AIP could be a valuable biomarker for the stratification of cognitive impairment risk, and maintaining lower AIP levels may help prevent cognitive impairment.

Cognitive decline has become a significant public health challenge globally, posing serious threats to both individual well-being and broader health outcomes (Alzheimer’s Association, 2015; Llibre Rodriguez et al., 2008; Roberts and Knopman, 2013). Cognitive impairment can emerge years before the clinical onset of dementia, yet effective therapeutic options to reverse this decline remain limited. Thus, there is an urgent need to determine the mechanisms and risk factors underlying cognitive impairment. Early identification of individuals at high risk, combined with timely and targeted interventions, is critical in mitigating the onset and progression of cognitive decline. Proactive measures in this respect are not only essential for delaying disease progression but also for reducing the substantial burden placed on healthcare systems and families.

Several studies have established a connection between metabolic disorders and cognitive impairment. A study in Korea, focusing on individuals aged 65 and older, identified metabolic disorders as an independent risk factor for cognitive impairment (Lee et al., 2017). Similarly, a systematic review of prospective cohort studies revealed that individuals with metabolic syndrome face a 1–2 times higher risk of cognitive decline compared to those without metabolic issues (Hao et al., 2011). A Chinese study further supported this by reporting a significant correlation between metabolic disorders and cognitive impairment (Wang et al., 2019). Among the components of metabolic disorders, dyslipidemia has emerged as a particularly important risk factor for cognitive impairment (Bai et al., 2024; Farr et al., 2008; Feinkohl et al., 2019; Kivipelto and Solomon, 2006; Ma et al., 2017). Elevated TG levels have been specifically associated with cognitive impairment, with research linking higher TG levels to cognitive impairment in various neurological and psychiatric conditions (Farr et al., 2008; Gonzalez-Escamilla et al., 2016; Lindenmayer et al., 2012). Recent findings suggest that elevated TG levels can negatively affect memory performance in middle-aged and older adults (Leritz et al., 2016), and TG levels are notably higher in older individuals with mild cognitive impairment compared to those without cognitive issues (Yao et al., 2016). These results suggest that TG levels are closely linked to cognitive impairment. In contrast, low levels of HDL-C have been identified as an independent risk marker for cognitive impairment in older adults (Feinkohl et al., 2019). HDL-C, commonly known as “good” cholesterol, is widely recognized for its protective effects on brain health (Ito and Ito, 2020). A recent study found a positive association between white matter volume and HDL-C levels, indicating that higher HDL-C levels may benefit cognitive function (Livingston et al., 2020). The AIP, calculated as the logarithmic ratio of triglycerides to HDL-C, serves as a sensitive marker of lipid and lipoprotein profiles (Dobiásová and Frohlich, 2001) and is considered a more reliable indicator of dyslipidemia than TG or HDL-C levels alone (McLaughlin et al., 2003; Onat et al., 2010; Zhu et al., 2015). It has also been strongly linked to various other conditions, including cardiovascular diseases, diabetes, and other metabolic disorders (Jiang et al., 2024; Qu et al., 2024).

Building on these findings, we conducted a follow-up observational study using data from the CHARLS database, focusing on middle-aged and older individuals. Taking advantage of the longitudinal cohort data, we gathered information from the 2018 cognitive function assessments to explore the relationship between AIP levels and cognitive impairment. The results revealed a significant positive association between baseline AIP levels and cognitive impairment in individuals aged 45 and above in China. The highest AIP quartile was associated with a 68% increased risk of cognitive impairment compared to the lowest quartile. Furthermore, the P for trend across AIP quartiles was <0.001 for all models, suggesting a significant and consistent trend, reinforcing a dose-response relationship between AIP levels and cognitive impairment risk. Although these effect sizes are moderate, they are clinically meaningful given the progressive nature of cognitive impairment, with even moderate increases in risk having important public health implications. Subgroup analysis, stratified by factors such as age and gender, also yielded consistent outcomes. Thus, AIP has the potential to be a novel lipid metabolism biomarker for cognitive impairment. And there is a non-linear relationship between AIP levels and the risk of cognitive impairment. The risk of cognitive impairment demonstrated a clear increase with higher AIP levels below the median value of 0.337. However, beyond this threshold, the rate at which the risk of cognitive impairment escalates appeared to slightly decelerate. This non-linear relationship may be attributed to the presence of additional comorbidities, such as cardiovascular diseases, diabetes, and chronic inflammation, which become more prominent as AIP levels increase. These factors could contribute to the attenuation of the direct impact of AIP on cognitive impairment, thereby leading to a less pronounced increase in cognitive impairment risk at higher AIP levels.

The underlying mechanisms linking the AIP and cognitive impairment are complex and remain incompletely understood, though several potential pathways may explain this association. One widely accepted explanation is that the relationship between lipids and cognitive function arises from dyslipidemia being a risk factor for stroke and cerebral hypoperfusion, both of which are closely linked to cognitive impairment (Wendell et al., 2009; Wright et al., 2008). Additionally, elevated cholesterol levels may have a more direct effect on cognitive function. While high cholesterol is involved in synaptogenesis and may initially aid in compensatory neural repair in the context of cognitive impairment (Mauch et al., 2001), it also contributes to the accumulation of β-amyloid peptides, which accelerates the progression of cognitive decline (Burns and Duff, 2002). Moreover, recent studies have identified the AIP as a robust biomarker of dyslipidemia, with higher levels suggesting more severe insulin resistance (IR) (Shi and Wen, 2023; Yin et al., 2023). There is also increasing evidence of a strong connection between IR and a heightened risk of cognitive decline (Hong et al., 2021; Hooshmand et al., 2019; Neergaard et al., 2017). IR is a distinctive metabolic disorder, typically characterized by elevated insulin levels. Prolonged exposure of brain neurons to elevated insulin may lead to neurodegeneration and persistent memory impairment (Blázquez et al., 2014). Additionally, IR can reduce glucose metabolism in specific regions of the brain, potentially adversely affecting memory function (Willette et al., 2015). Several clinical studies have suggested that TG can cross the blood-brain barrier, potentially impairing cognitive function by inducing insulin receptor resistance (Banks et al., 2018). Moreover, the AIP not only reflects elevated TG levels but also indicates reduced HDL-C levels. Low HDL-C may diminish its neuroprotective effects, such as anti-inflammatory actions and the inhibition of β-amyloid aggregation, while high TG levels can further damage neurons, exacerbating cognitive decline (Cockerill et al., 1995; Cockerill et al., 2001; McGeer and McGeer, 1998). TG levels may impair cognition through several mechanisms. TG metabolites can influence glutamate release and alter N-methyl-D-aspartate receptor-mediated calcium influx, potentially disrupting synaptic plasticity in the hippocampus. Additionally, TG-induced oxidative stress and chronic inflammation may damage neuronal signaling. Elevated TG levels could also hinder leptin from crossing the blood-brain barrier, thereby reducing its cognitive-enhancing effects (Chang et al., 2004; McGeer and McGeer, 1998).

Cognitive impairment has become an increasingly prominent issue, strongly associated with poor outcomes and significantly impacting individuals’ quality of life, while also placing a considerable burden on families and society. The early prevention of cognitive impairment is thus of critical importance. Our findings offer valuable clinical insights into reducing the incidence of cognitive impairment. Our study specifically found that the AIP holds significant clinical value as a predictor of cognitive impairment in the Chinese population aged 45 years and older. Because the AIP is calculated from routine lipid measurements (TG and HDL-C), it is a simple, cost-effective marker that accurately reflects an individual’s lipid metabolism. Our results indicate a clear positive correlation between elevated AIP levels and the risk of developing cognitive impairment, suggesting that the AIP may serve as a practical and reliable lipid-based biomarker for predicting cognitive decline. The clinical advantages of the AIP include its ease of measurement, reproducibility, and robust predictive capacity. Compared to other more complex or less widely applicable biomarkers, the AIP, as part of standard lipid testing, offers the potential for large-scale screening and early intervention. As such, the AIP has the potential to be a valuable tool in clinical practice for assessing cognitive impairment risk, facilitating the early identification of high-risk individuals, and enabling timely preventive measures. However, further rigorous and large-scale prospective studies are needed to confirm the clinical utility of the AIP as an early predictor of cognitive impairment and to establish its applicability across different populations.

This study has several limitations. First, as an observational study, although we attempted to control for known confounders, unmeasured confounding factors may still have influenced the results. As our study relied on existing data from CHARLS, the availability of certain variables was limited, which may have further contributed to unmeasured confounding. Secondly, we only examined the impact of baseline AIP levels from 2011 on subsequent cognitive impairment, without considering the potential effects of dynamic changes in the AIP over time on cognitive function. Future studies incorporating repeated measurements of AIP and its contributing lipid components would allow for a more comprehensive assessment of its long-term impact on cognitive health and help determine whether cumulative exposure or temporal changes in AIP play a significant role in cognitive impairment. And participant attrition may introduce bias and affect the generalizability of the findings. However, we have made adjustments for baseline characteristics and potential confounders to minimize this impact. Additionally, test-retest reliability for the cognitive measures has not been reported in the CHARLS dataset. while we evaluated cognitive function using episodic memory and mental acuity—reliable screening tools widely used in clinical practice—a more comprehensive assessment across multiple cognitive domains would be necessary to fully understand overall cognitive function. Moreover, the study did not differentiate between the underlying causes of cognitive impairment, and all participants were from China. Future research should aim to expand the scope of data collection to include a more diverse range of participants and conduct more detailed analysis of different types of cognitive impairment across various ethnic groups to strengthen our findings.

## 5 Conclusion

The study demonstrates that elevated AIP levels are associated with an increased risk of cognitive impairment in middle-aged and older adults, showing a non-linear dose-response relationship between the AIP and cognitive function. This suggests that targeted interventions aimed at managing dyslipidemia may play a beneficial role in reducing the incidence of cognitive impairment.

## Data Availability

Publicly available datasets were analyzed in this study. This data can be found here: the data is sourced from the publicly accessible CHARLS database (http://charls.pku.edu.cn/). The datasets used during the current study are available from the corresponding author on reasonable request.

## Footnotes

1
http://charls.pku.edu.cn/
